# Knowledge, Attitudes, and Perceptions Regarding Mini Implants Among Orthodontic Practitioners in the Kurdistan Region: Cross-Sectional Study

**DOI:** 10.2196/86896

**Published:** 2026-02-26

**Authors:** Soran M Hameed, Aras M Rauf

**Affiliations:** 1 Orthodontic Training Center Sulaymaniyah, Kurdistan Region Iraq; 2 Department of Dentistry College of Dentistry University of Sulaimani Sulaminyay, Kurdistan Region Iraq

**Keywords:** orthodontic mini implants, temporary anchorage devices, orthodontists, knowledge, attitude, perception, skeletal anchorage, Kurdistan Region, cross-sectional study

## Abstract

**Background:**

Mini implants, or temporary anchorage devices, have transformed modern orthodontic practice by offering stable, minimally invasive anchorage for complex tooth movements. Despite their proven effectiveness, their use varies widely across regions, often influenced by clinicians’ knowledge, confidence, and training.

**Objective:**

This study explored the knowledge, attitudes, and perceptions of orthodontic professionals regarding mini implant use in the Kurdistan Region, Iraq, and examined how experience and professional background shape their adoption in daily clinical work.

**Methods:**

A cross-sectional online survey was conducted between April 2025 and September 2025 among orthodontic faculty and postgraduate trainees from 7 dental colleges in the Kurdistan Region. The validated questionnaire assessed participants’ demographic details and 3 key domains—knowledge, perceptions, and attitudes—using Likert-scale responses. Data were analyzed using SPSS (version 28.0) and the Mann-Whitney *U*, Kruskal-Wallis, and Spearman correlation tests, with significance set at *P*≤.05.

**Results:**

A total of 175 orthodontic practitioners completed the survey (n=100, 57.1% postgraduate trainees; n=75, 42.9% faculty members). Postgraduate trainees demonstrated significantly higher knowledge (mean 3.66, SD 0.49 vs mean 3.16, SD 0.48; *P*=.01) and perception (mean 3.29, SD 0.60 vs mean 2.39, SD 0.58; *P*=.02) scores. Immediate loading was preferred by 80% (80/100) of the postgraduate trainees compared with 40% (30/75) of faculty members (*P*=.001), whereas radiographic guidance was selected as the safest placement method by 75% (75/100) of postgraduate trainees vs 40% (30/75) of faculty members (*P*<.001). Younger clinicians (aged <35 years) and those with less than 5 years of experience showed significantly higher perception scores (*P*=.01). Knowledge, perception, and attitude were strongly correlated (*r*=0.74; *P*<.001), indicating that increased understanding promotes more positive attitudes toward mini screw use.

**Conclusions:**

Orthodontists in the Kurdistan Region generally hold favorable views toward mini implants, yet differences in confidence and hands-on experience remain evident across generations.

## Introduction

Orthodontic implants trace their origins to the mid-20th century, when Gainsforth and Higley [1] first described the use of screws as auxiliary anchorage devices for tooth movement. Although their initial attempts achieved limited implant survival, these pioneering experiments laid the foundation for future advancements. Over the subsequent decades, orthodontic research focused intensively on developing reliable intraoral anchorage systems, leading to significant innovations in implant design and application for both prosthodontic and orthognathic purposes [2].

The evolution of mini screw technology accelerated in the 1990s, when Kanomi [3] introduced screws specifically designed for orthodontic use. Improvements in design geometry, size, and alloy composition enhanced both biocompatibility and mechanical stability, making mini screws more accessible and predictable for routine clinical practice. Several specialized systems have emerged, including the Aarhus mini implant, Spider Screw, and AbsoAnchor microimplant, reflecting global efforts to optimize skeletal anchorage [4].

Achieving optimal anchorage control remains one of the most persistent challenges in orthodontics, directly influencing treatment predictability and success. The introduction of skeletal anchorage systems, particularly orthodontic mini screws, has represented a paradigm shift in anchorage reinforcement, offering a stable, compliance-free alternative for complex tooth movements. Mini implants are appreciated for their versatility, minimal invasiveness, ease of placement and removal, and capacity for immediate or early loading. These advantages have broadened their indications across numerous nonsurgical orthodontic applications such as en masse retraction, molar distalization, open bite correction, maxillary expansion, and the management of complex malocclusions. Clinical success is primarily linked to mini screw stability, which depends on bone quality, insertion torque and depth, and biomaterial characteristics. Reported survival rates often exceed 80% to 90%, affirming their reliability as adjuncts in contemporary orthodontic treatment [2].

Despite these benefits, the global adoption of mini implants remains uneven, influenced by practitioners’ knowledge, clinical experience, and access to specialized training. Reported barriers include screw loosening (up to 83%), soft tissue irritation (59.7%), procedural failure, and concerns about patient comfort and oral hygiene. These factors underscore the ongoing need for technical proficiency, standardized guidelines, and practitioner confidence [5,6].

Recent studies show that orthodontists’ willingness to use mini screws is shaped by variables such as professional experience, continuing education, gender, and practice setting. A study revealed that over 90% of orthodontists who use mini screws report satisfactory or highly satisfactory treatment outcomes, yet concerns about cost, procedural complexity, and potential complications persist. Clinical factors rather than patient demographics such as age or gender are the primary determinants of mini screw success [7,8].

Worldwide, the use of mini screws continues to expand, supported by strong clinical evidence and favorable outcomes. However, a gap remains between early-career and senior orthodontists regarding optimal placement sites, complication management, and biomechanical integration. Addressing these disparities requires structured education, mentorship, and evidence-based training to support effective diffusion of this technology [9,10].

In response to these trends, this study investigated the current knowledge, attitudes, and perceptions (KAP) related to orthodontic mini screw use among orthodontists in the Kurdistan Region, Iraq. This study also examines the demographic and professional factors influencing clinicians’ readiness to incorporate mini screws into routine practice. It is hypothesized that there will be significant variation in KAP scores based on experience level, training background, practice environment, and access to professional development opportunities. Specifically, this research aimed to (1) assess orthodontists’ knowledge of mini screw indications, benefits, and complications; (2) evaluate their actual clinical practices, including preferred insertion sites, perceived success rates, and common barriers; (3) explore attitudes toward mini screws compared with conventional anchorage methods; and (4) identify the major educational and technical gaps limiting their optimal use.

This investigation represents the first comprehensive regional evaluation of orthodontists’ readiness to adopt mini implant technology, with findings expected to inform targeted continuing education programs, enhance clinical quality, and advance evidence-based orthodontic care across the Kurdistan Region.

## Methods

### Study Design and Setting

This cross-sectional, descriptive, questionnaire-based study was conducted from April 1, 2025, to September 1, 2025, by the Medical Research Scientific and Ethical Committee in collaboration with orthodontic faculty and postgraduate practitioners from dental colleges across the Kurdistan Region of Iraq. Eligible participants included registered orthodontists in the Kurdistan Dental Association and postgraduate trainees actively studying in accredited dental institutions within the region.

### Ethical Considerations

The study protocol was approved by the Kurdistan Higher Council of Medical Specialties Medical Research Scientific and Ethical Committee. It was conducted in accordance with the ethical principles of the 2013 Declaration of Helsinki. All participation was entirely voluntary, and the study objectives and confidentiality safeguards were clearly stated before participation. Informed consent was explicitly obtained before the start of the survey. Participants did not receive any compensation for taking part in the study. To protect participants’ anonymity, the survey did not collect any personally identifying information, and all responses were accessible solely to the principal investigator.

### The Questionnaire

#### Overview

The questionnaire was constructed following the CHERRIES (Checklist for Reporting Results of Internet E-Surveys; Typeform) guidelines, ensuring concise, validated, and ethically sound data collection. The first section of the questionnaire included a demographic statement detailing the study objectives, eligibility criteria, voluntariness of participation, and contact details of the principal investigator.

The questionnaire was systematically structured into 3 consecutive sections (A, B, and C) to ensure that, upon completion of the study, a comprehensive assessment and understanding could be achieved regarding the respondents’ characteristics and their knowledge about, perceptions of, and clinical attitudes toward orthodontic mini screws.

#### Demographic Section

This section collected information on participants’ age, gender, academic designation, region of practice, years of experience, and involvement in clinical orthodontic work.

#### Knowledge Assessment

This subsection included multiple-choice and Likert-scale items that evaluated participants’ understanding of mini implant indications, benefits, complications, loading protocols, and stability principles.

#### Perception Assessment

Perception was assessed using Likert-scale items measuring participants’ views on clinical safety, barriers to use, success rates, loading preferences, and confidence regarding mini screw application.

#### Attitude Assessment

Attitude items explored respondents’ willingness to use mini implants, openness to adopting new techniques, preferred clinical approaches, and readiness for training and skill development.

### Pretesting and Content Validity of the Questionnaire

The questionnaire was developed through the collaborative input of a panel consisting of 2 orthodontists, 1 academic statistician, and 1 researcher with over 15 years of research experience, all independent from the study’s participant pool. Content validity was established via expert review, with the Aiken *V* statistic determined to be 0.89, indicating a high level of agreement among reviewers. To further refine the instrument, a pilot test was conducted with 25 orthodontists and postgraduate trainees not included in the final sample.

The internal consistency of the questionnaire was assessed using the Cronbach α, yielding coefficients of 0.85 for the knowledge domain and 0.91 for attitude and perception items. Test-retest reliability was evaluated by readministering the instrument to the pilot group after a 3-week interval, resulting in an interobserver κ coefficient of 0.88. These procedures ensured strong measurement validity and reliability, with the finalized version distributed to a sample of approximately 120 orthodontists and postgraduate participants, aligning with standard methodological frameworks for cross-sectional KAP surveys in dental research.

### Sample Size Estimation

The sample size was estimated using the online tool provided by the Calculator website [11], ensuring an evidence-based approach for determining the appropriate participant number [12]. According to records from the Kurdistan Dental Association, the total number of orthodontists and postgraduate trainees practicing in the region was approximately 210 at the time of the study. Using this population figure, a statistical power analysis was conducted to calculate the minimum required sample size.

Assuming an anticipated response proportion of 50% on mini screw–related knowledge and attitudes with a 5% margin of error and a confidence level set at 95%, the recommended minimum sample size was 132. Considering an estimated response rate of 75% based on prior regional survey experiences, the questionnaire was distributed to 175 practitioners to achieve a representative and statistically robust sample.

### Study Participants and Data Collection Procedure

The roster of dental institutions and relevant contact details were obtained from the official records of the Kurdistan Dental Association and the Ministry of Higher Education and Scientific Research of the Kurdistan Region. Formal correspondence, endorsed by the head of the orthodontics department, was sent to dental colleges and teaching hospitals across the northern provinces requesting updated lists of both orthodontic faculty and postgraduate trainees, including email and telephone contact information. Among the 7 dental colleges in the Kurdistan Region (with representation from Erbil, Sulaymaniyah, Duhok, Halabja, and the surrounding areas), a total of 123 postgraduate orthodontic trainees and 87 faculty members were identified, for a combined total of 210 eligible participants—a postgraduate trainee–to–faculty member ratio of approximately 1.4:1.

A convenience nonprobabilistic sampling approach was used. Google Form invitations, including instructions and electronic consent, were distributed proportionally to 175 individuals (n=100, 57.1% postgraduate students and n=75, 42.9% faculty members) via email and professional WhatsApp groups, reflecting the regional distribution and the desired sample size as determined via power analysis. Participants had the opportunity to complete the questionnaire once. Duplicate and incomplete responses were screened out, and reminder messages were sent 2 weeks after the initial invitation to optimize the response rate. Inclusion was limited to orthodontic faculty and postgraduate trainees, whereas undergraduate students, those who did not provide consent, and incomplete submissions were excluded from the study.

### Scoring Criteria

#### Overview

In the analysis, each Likert-scale question except for designated demographic and multiple-choice questions was scored as follows: “agree” was assigned a value of 1, “neutral” was assigned a value of 0, and “disagree” was assigned a value of −1. For the knowledge section, comprising 8 Likert-scale items, the maximum achievable score was 8. Similarly, the perception and attitude sections each contained 6 Likert-scale items, yielding maximum possible scores of 6 for each domain. Summary metrics, including means and SDs, were calculated for the knowledge, perception, and attitude scores to enable quantitative comparison among respondents.

#### Knowledge Scoring

Knowledge items were scored using a 3-point Likert scale where “agree”=1, “neutral”=0, and “disagree”=−1.

The knowledge section contained 8 items, yielding a possible score range of −8 to +8, with higher scores indicating better knowledge regarding mini screw use.

#### Perception Scoring

Perception items were also scored using the same Likert scale (“agree”=1, “neutral”=0, and “disagree”=−1).

This section included 6 items, allowing for a maximum perception score of +6.

Higher values reflected a more positive perception of mini screw application.

#### Attitude Scoring

Attitude items followed the same scoring pattern (1, 0, and −1). Six attitude items were included, with a maximum total possible score of +6. Higher scores indicated a more favorable attitude toward incorporating mini screws into orthodontic practice.

### Statistical Analysis

Data from the online questionnaire platform were checked and extracted. Any apparent disparity or error was spotted, discussed, and reconciled to ensure that incomplete or inconsistent entries were successfully eliminated from the final analysis. The SPSS software (version 28.0; IBM Corp) was used for the statistical analyses.

The normality of continuous variables was checked via the Shapiro-Wilk test. Descriptive statistics such as frequencies and percentages were used to summarize sociodemographic characteristics and the distribution of responses. Group comparisons applied nonparametric tests: the Mann-Whitney *U* test for dichotomous variables (such as gender) and the Kruskal-Wallis test for multigroup comparisons by experience level, academic position, or region. Where significant differences emerged, Dunn post hoc tests were used in pairwise analyses. KAP scores were also analyzed using these nonparametric methods. Associations between ordinal variables such as KAP scores and years of experience were tested using the Spearman rank-order correlation coefficient (Spearman ρ). The threshold for statistical significance was set at *P*≤.05 across all analyses.

## Results

### Participant Demographics

Table 1 shows the baseline demographic characteristics of the study respondents. Of the total sample size of 175 valid responses, 100 (57.1%) were from postgraduate students, whereas 75 (42.9%) were faculty members. Participants were recruited from different provinces, and their distribution across Kurdistan dental colleges varied by region. The respondent population was balanced between men and women, and most were in the age group of 26 to 35 years. A total of 66.9% (117/175) indicated less than 5 years of professional experience, and 12.6% (22/175) indicated more than 10 years of professional experience. The study sample represented orthodontic practitioners from several provinces in the Kurdistan Region, with participants from Erbil, Sulaymaniyah, and Duhok, reflecting a diverse subregional distribution. A total of 87% (65/75) of the faculty members were in private practice.

**Table 1:** Baseline characteristics of the study participants (n=175).

Demographic variable and category		Participants, n (%)
**Age group (y)**		
	<25	29 (16.6)
	26-35	108 (61.7)
	36-45	29 (16.6)
	>45	9 (5.1)
**Sex**		
	Male	88 (50.3)
	Female	87 (49.7)
**Academic designation**		
	Postgraduate students	100 (57.1)
	Faculty members	75 (42.9)
**Experience (y)**		
	<5	117 (66.9)
	5-10	36 (20.6)
	>10	22 (12.6)
**Private practice (faculty only; n=75)**		
	Yes	65 (86.7)
	No	10 (13.3)

### Knowledge Results

Significant differences in knowledge-related items were observed between the 2 groups. For primary stability mechanisms, 60% (60/100) of postgraduate students attributed mini screw stability to bone quality, whereas 55% (41/75) of faculty members emphasized cortical engagement (*P*=.002).

Regarding success rates, 70% (70/100) of postgraduate students reported 80% to 90% success rates, whereas 65% (49/75) of faculty members reported success rates above 90% (*P*=.03).

For clinical indications, anterior retraction was selected by 55% (55/100) of postgraduate students compared with 27% (20/75) of faculty members, whereas molar intrusion or extrusion was selected by 45% (34/75) of faculty members (*P*=.02). With respect to placement safety, 75% (75/100) of postgraduate students preferred radiographic imaging, whereas 55% (41/75) of faculty members favored safe angulation techniques (*P*<.001). A detailed comparison of knowledge-related responses is shown in Table 2.

**Table 2:** Comparison of responses between postgraduate students and faculty members using the chi-square test.

Variable and response		Postgraduate students (n=100), n (%)	Faculty members (n=75), n (%)	Chi-square (*df*)		*P* value	
**Primary stability mechanism**					9.4 (2)		.002
	Bone quality and density	60 (60)	26 (34.7)				
	Cortical engagement	30 (30)	41 (54.7)				
	Mechanical interlocking	10 (10)	8 (10.7)				
**Success rate (%)**					7.1 (2)		.03
	80-90	70 (70)	22 (29.3)				
	>90	20 (20)	49 (65.3)				
	<80	10 (10)	4 (5.3)				
**Clinical indication**					5.9 (2)		.02
	Anterior retraction	55 (55)	20 (26.7)				
	Molar intrusion or extrusion	25 (25)	34 (45.3)				
	Others	20 (20)	21 (28)				
**Placement safety**					15.2 (2)		<.001
	Radiographic imaging	75 (75)	30 (40)				
	Safe angulation	20 (20)	41 (54.7)				
	Others	5 (5)	4 (5.3)				
**Loading protocol**					10.8 (1)		.001
	Immediate	80 (80)	30 (40)				
	Delayed (4-6 wk)	20 (20)	45 (60)				
**Frequency of TAD^a^** **use**					20.5 (1)		<.001
	Rarely or sometimes	70 (70)	15 (20)				
	Often or routinely	30 (30)	60 (80)				
**Clinical applications**					1.8 (1)		.18
	Anterior retraction	50 (50)	36 (48)				
	Others	50 (50)	39 (52)				
**Operator preference**					0.7 (1)		.41
	Orthodontist	80 (80)	65 (86.7)				
	Others	20 (20)	10 (13.3)				
**Anchorage preference**					0.5 (1)		.62
	Direct anchorage	55 (55)	36 (48)				
	Indirect or hybrid	45 (45)	39 (52)				

^a^TAD: temporary anchorage device.

### Perception Results

Significant differences in perception-related items were observed between postgraduate trainees and faculty members. Postgraduate participants showed a stronger preference for radiographic imaging as the safest approach for mini screw placement, with 75% (75/100) selecting this option, whereas 55% (41/75) of faculty members predominantly favored safe insertion angulation techniques (*P*<.001).

Perceptions regarding loading protocols also varied notably: immediate loading was preferred by 80% (80/100) of postgraduate trainees, whereas delayed loading after 4 to 6 weeks was favored by 60% (45/75) of faculty members (*P*=.001). With respect to frequency of use, 70% (70/100) of postgraduate students reported rarely or occasionally using mini screws, whereas 80% (60/75) of faculty members indicated routine use (*P*<.001).

Despite these differences, perceptions related to clinical operator preference and anchorage type showed no statistically significant variation between groups, indicating general agreement in these areas. A detailed comparison of perception-related responses is shown in Table 2.

### Attitude Results

Attitude-related findings indicated generally positive views on the clinical use of mini screws among both postgraduate trainees and faculty members. Operator preference was consistent across groups, with 80% (80/100) of postgraduate students and 87% (65/75) of faculty members indicating that mini screw placement should be performed by orthodontists, showing no statistically significant differences.

Similarly, attitudes toward anchorage type exhibited comparable patterns. Direct anchorage was selected by 55% (55/100) of postgraduate students and 48% (36/75) of faculty members, whereas indirect or hybrid anchorage approaches were reported at nearly equal rates across both groups, suggesting shared clinical attitudes toward anchorage selection.

In addition, several barriers influencing clinicians’ attitudes toward broader adoption of mini screws were highlighted. These included concerns related to procedural safety, operator experience, and treatment cost, all of which contributed to variations in clinicians’ readiness and confidence to integrate mini screws into routine orthodontic practice.

### KAP Score Comparison

Table 3 shows that the mean KAP scores for male and female participants did not vary with any statistically significant difference. However, postgraduate students recorded significantly higher mean knowledge and perception scores when compared to faculty members; the attitudes between both groups were comparable. Among faculty, senior lecturers obtained somewhat higher scores than professors in perception, although this difference fell short of being statistically significant. When experience was considered, it was found that those participants who had less than 5 years of practice obtained the highest perception scores (mean 3.10, SD 0.85), which were significantly different from those of individuals with more than 10 years of experience (mean 2.62, SD 0.90). With respect to age groups, while the youngest group—26 to 35 years—attained the maximum knowledge and perception scores, the lowest mean scores were obtained in the age group of 46 to 50 years. Post hoc analysis further revealed statistically significant differences in attitude scores between the age group of 26 to 35 years and both the age groups of 36 to 45 years and 46 to 50 years.

**Table 3:** Mean knowledge, perception, and attitude scores with respect to age, gender, academic designation, and years of experience.

Variable and category			Number of participants	Knowledge score (–8 to +8), mean (SD)	*P* value		Perception score (–6 to +6), mean (SD)			*P* value		Attitude score (–6 to +6), mean (SD)		*P* value	
**Sex**						.81					.65				.51
	Male		95	3.15 (0.70)			2.87 (0.66)					1.92 (0.57)			
	Female		80	3.20 (0.68)			2.79 (0.62)					2.05 (0.59)			
**Academic designation**						.01^a^					.02^a^				.73
	Postgraduate students		100	3.66 (0.49)			3.29 (0.60)					2.04 (0.48)			
	**Faculty members**		75	3.16 (0.48)	.15		2.39 (0.58)			.14		1.98 (0.50)		.63	
		Professors	20	3.10 (0.50)					2.35 (0.52)			1.92 (0.46)			
		Senior lecturers	30	3.25 (0.47)					2.55 (0.53)			2.02 (0.44)			
		Readers	25	3.18 (0.52)					2.45 (0.55)			2.00 (0.48)			
**Experience (y)**						.04^a^					.01^a^				.48
	<5		62	3.68 (0.47)			3.10 (0.85)					1.95 (0.71)			
	5-10		50	3.42 (0.53)			2.75 (0.82)					2.12 (0.52)			
	>10		63	3.18 (0.55)			2.62 (0.90)					2.05 (0.60)			
**Age (y)**						.01^a^					.02^a^				.02^a^
	26-35		85	3.43 (0.48)			3.05 (0.62)					1.96 (0.58)			
	36-40		38	3.12 (0.51)			2.75 (0.65)					2.22 (0.57)			
	41-45		22	2.95 (0.55)			2.62 (0.68)					2.04 (0.53)			
	46-50		15	2.90 (0.60)			2.10 (0.70)					1.87 (0.54)			
	51-55		15	3.43 (0.48)			2.83 (0.64)					2.16 (0.42)			

^a^Considered significant at *P*<.05.

### Correlation Between KAP and Demographic Variables of the Participants

Table 4 shows that the KAP scores of participants varied negatively though weakly with age, years of experience, and designation. In other words, older participants, those with more than 10 years of clinical experience, and professors obtained relatively lower scores than younger individuals, those with fewer years of experience, and postgraduate students. These differences were statistically significant, particularly for perception scores. However, on the other hand, designation and years of experience showed a strong positive correlation with age. This means that, as the participants age, they hold higher academic positions and have a longer duration of professional practice. Most importantly, the correlations among knowledge, perception, and attitude scores were strongly positive; as knowledge improved, the perception and attitudes regarding the use of temporary anchorage devices (TADs) in orthodontics also improved.

**Table 4:** Correlation between knowledge, attitude, and perception scores and age, academic designation, and years of experience of the participants using the Spearman correlation coefficient test.

Variables			Age		Knowledge		Perception	Attitude		Academic designation		Experience
**Age**												
	*r*	1		−0.12		−0.15^a^		−0.08	0.42^b^		0.55^c^	
	*P* value	—^d^		.09		.04^e^		.19	<.001^e^		<.001^e^	
**Knowledge score**												
	*r*	−0.12		1		0.74^c^		0.46^b^	−0.10		−0.18^a^	
	*P* value	.09		—		<.001^e^		.004^e^	.24		.03^e^	
**Perception score**												
	*r*	−0.15^a^		0.74^c^		1		0.51^b^	−0.14^a^		−0.20^a^	
	*P* value	.04^e^		<.001^e^		—		.002^e^	.03^e^		.02^e^	
**Attitude score**												
	*r*	−0.08		0.46^b^		0.51^b^		1	−0.11		−0.09	
	*P* value	.19		.004^e^		.002^e^		—	.21		.16	
**Academic designation**												
	*r*	0.42^b^		−0.10		−0.14^a^		−0.11	1		0.51^c^	
	*P* value	<.001^e^		.24		.03^e^		.21	—		<.001^e^	
**Experience**												
	*r*	0.55^c^		−0.18^a^		−0.20^a^		−0.09	0.51^c^		1	
	*P* value	<.001^e^		.03^e^		.02^e^		.16	<.001^e^		—	

^a^Negative *r* values indicate a negative correlation (*r*=0.10-0.29 indicates a weak correlation).

^b^*r*=0.30 to 0.49 indicates a moderate correlation.

^c^*r*≥0.50 indicates a strong correlation.

^d^Not applicable.

^e^Considered significant at *P*<.05.

## Discussion

### Principal Findings

This study found that postgraduate trainees exhibited significantly higher knowledge and perception scores than faculty members, whereas attitudes were generally positive across both groups. Younger participants and those with fewer years of experience also showed more favorable views on mini screw use. This study also offers valuable insights into the awareness, perceptions, and clinical perspectives surrounding the use of orthodontic mini screws (TADs) among postgraduate trainees and academic faculty in the Kurdistan Region of Iraq. The findings reflect a pattern consistent with that found in the global literature, highlighting both notable progress and persisting challenges in integrating mini screws into routine orthodontic practice.

The comparative analysis revealed significant differences in foundational knowledge and preferred clinical protocols between postgraduate trainees and faculty members. Postgraduate trainees achieved higher knowledge and perception scores and tended to favor immediate loading, radiographic evaluation for safety, and direct anchorage approaches. In contrast, faculty members preferred delayed loading and placed greater emphasis on safe insertion angulation. These variations have been well documented in previous research. For instance, Panaite et al [5] reported that both clinical experience and structured postgraduate training strongly influence the successful adoption and clinical mastery of mini screws. Nonetheless, the absence of consistent, hands-on education remains a global barrier to optimal application. Similarly, Al-Hammadi [13] identified insufficient training as a primary obstacle to broader adoption across the Middle East and Asia, a challenge that extends beyond Iraq.

The barriers identified in this study (operator experience, placement safety, and procedural cost) align closely with findings from China, Saudi Arabia, Canada, and Europe. A persistent training gap, compounded by perceived procedural complications, continues to slow widespread clinical integration. Reviews by Ahmed et al [14] and Panaite et al [5] further highlighted concerns regarding fracture resistance, optimal insertion protocols, and management of mini screw failure. However, recent evidence emphasizes that, when clinicians are trained through evidence-based protocols, mini screws consistently deliver predictable mechanical retention across a range of orthodontic scenarios [15,16].

The data from this study revealed a strong positive correlation among knowledge, perception, and attitude scores. In other words, as educational exposure increased, so did awareness, confidence, and willingness to incorporate mini screws into clinical practice [17]. Younger clinicians generally exhibited higher perception scores, reflecting their exposure to updated curricula and modern training environments. Senior faculty members, on the other hand, appeared more cautious, possibly due to established clinical routines and limited early exposure to TAD technology. This generational contrast underscores the importance of structured, continuous education at all professional levels [18].

Table 1 shows balanced participation between postgraduate trainees and faculty members, as well as between male and female respondents. Most (108/175, 61.7%) were aged 26 to 35 years, and nearly two-thirds (117/175, 66.9%) reported less than 5 years of professional experience. This demographic profile is consistent with similar regional and international studies, supporting the representativeness and reliability of this sample [5].

Table 2 compares the knowledge and clinical perceptions of postgraduate students and faculty members. Postgraduate students were more likely to attribute primary stability to bone quality, whereas faculty members emphasized cortical engagement—reflecting distinct experiential perspectives. Interestingly, faculty members reported higher perceived success rates (>90%), whereas postgraduate students estimated success within the 80% to 90% range. Statistically significant differences were found in preferred indications, safety protocols, loading strategies, and frequency of TAD use (*P*<.05). These outcomes align with existing literature indicating that accumulated experience often leads clinicians to adopt more conservative yet evidence-based techniques [19]. The general agreement between both groups regarding operator responsibility and anchorage principles suggests a shared understanding of fundamental clinical concepts.

Table 3 outlines the mean KAP scores across demographic variables. Gender was not a significant factor in any domain. Postgraduate students demonstrated significantly higher knowledge and perception scores than faculty members, although attitude scores were comparable between groups. Participants with less than 5 years of experience achieved the highest perception scores, whereas those with more than 10 years of practice recorded the lowest perception scores. Younger professionals (aged 26-35 years) also scored higher in both knowledge and perception. This trend aligns with international evidence indicating that early-career practitioners often exhibit greater enthusiasm and openness toward new technologies, including TADs, largely due to their exposure to evolving curricula and training systems [20].

Table 4 presents the correlations between demographic factors and KAP scores. Both age and years of experience exhibited weak but negative correlations with knowledge, perception, and attitude scores, meaning that older and more experienced clinicians tended to score lower in these areas. While this may seem counterintuitive, it is consistent with literature showing that senior practitioners often resist altering long-standing clinical approaches [21]. Nevertheless, the strong positive interrelationships among knowledge, perception, and attitude scores underscore that enhancing education and training can meaningfully improve clinician confidence and acceptance of mini screw use—an observation echoed across global research.

Overall, older participants, those with more than 10 years of professional experience, or those with senior academic ranks showed lower confidence, knowledge, and perceptions regarding mini screws than their younger counterparts. This generational divide likely stems from differences in educational exposure: senior clinicians were trained when skeletal anchorage concepts were not yet standard, whereas newer graduates have benefited from curricula and workshops incorporating TADs. Consequently, senior practitioners may be less inclined to modify established routines.

At the same time, differences in technological familiarity also shape these perspectives. Younger clinicians, more accustomed to digital resources and modernized learning platforms, tend to approach innovations such as mini screws with greater openness and confidence [21]. Encouragingly, many senior respondents expressed an interest in updating their skills and applying mini screw techniques in clinical practice. This willingness to engage in professional development offers a promising opportunity for improved uptake—particularly if supported through structured continuing education programs, mentorship models, and hands-on workshops tailored to different experience levels.

The findings of this study highlight a clear need to strengthen the educational infrastructure supporting the use of mini screws in orthodontic training and clinical practice. Integrating structured, evidence-based modules on TAD biomechanics, placement protocols, and complication management into postgraduate curricula could substantially improve clinician confidence and treatment outcomes. Additionally, tailored continuing education programs aimed at midcareer and senior orthodontists would help bridge the generational gap in knowledge and perception identified in this study.

Future research should focus on longitudinal assessments of how enhanced training and exposure influence clinical adoption and success rates of mini screws over time. Comparative studies across different institutions and regions in Iraq and broader Middle Eastern contexts could further elucidate contextual barriers to and enablers of technology uptake. Finally, mixed methods designs incorporating qualitative interviews may provide richer insights into clinicians’ motivations, hesitations, and learning experiences related to skeletal anchorage systems. Such efforts will not only advance clinical practice but also contribute to the global dialogue on innovation adoption and educational reform in orthodontics.

### Limitations

A review of the limitations of this study must include a note about the possibility of age bias. More recent concepts discussed in the questionnaire, such as newer techniques related to the use of mini screws, might not be known or attractive to older clinicians. Thus, the online mode of survey distribution (Typeform and other electronic platforms) may have contributed to sampling bias by favoring younger participants, who are more active on digital platforms. Our electronic survey tool, and specifically its implementation through Typeform, probably also favored more active younger professionals on digital platforms, hence causing an overrepresentation of early-career respondents.

The questionnaire was made in English as it is the language normally used for dental education and practice in this region. This posed a language barrier to those not very comfortable with the use of English, which could also mean that some people were left out. Likert scales were applied to closed-ended questions; although very easy to apply when collecting quantitative data, they may restrict the expression of different thoughts or detailed logic by respondents, hence introducing the possibility of misreading or oversimplification of varied attitudes and experiences. Reliance on electronic distribution platforms (email and professional WhatsApp groups) may have contributed to sampling bias by increasing participation among younger, technology-oriented clinicians.

### Recommendations

This study reveals that orthodontists have a positive perception of mini screws and want to use them more often. To break the barriers of limited experience and safety, it is proposed to introduce focused training on mini screws in the dental education system and carry out targeted workshops for already established clinicians. This may be implemented through annual regional hands-on workshops, continuing professional development courses, and collaborative training programs involving dental colleges, teaching hospitals, and professional orthodontic associations. This will improve the skills and confidence in the safe adoption of mini screw techniques at all stages of a career.

### Conclusions

Most participants appreciated the clinical advantages of mini screws and were ready to accept them as potential good anchorage tools. This study inferred that improving the availability of advanced technical resources in dental clinics accompanied by oriented education at the undergraduate and postgraduate levels can play a role in solving the existing hindrances against the wider application of mini screw techniques in orthodontic practice.

## Abbreviations

CHERRIES: Checklist for Reporting Results of Internet E-Surveys
KAP: knowledge, attitudes, and perceptions
TAD: temporary anchorage device

## Data Availability

The datasets generated or analyzed during this study are available from the corresponding author on reasonable request.
